# Evaluation of Changes in Circulating Cell-Free DNA as an Early Predictor of Response to Chemoradiation in Rectal Cancer—A Pilot Study

**DOI:** 10.3390/medicina59101742

**Published:** 2023-09-28

**Authors:** Wee Liam Ong, Sorinel Lunca, Stefan Morarasu, Ana-Maria Musina, Alina Puscasu, Stefan Iacob, Irina Iftincai, Andreea Marinca, Iuliu Ivanov, Cristian Ena Roata, Natalia Velenciuc, Gabriel Dimofte

**Affiliations:** 1 2nd Department of Surgical Oncology, Regional Institute of Oncology (IRO), 700483 Iasi, Romania; william05021990@gmail.com (W.L.O.); musina.anamaria@gmail.com (A.-M.M.); alinaioanapuscasu@yahoo.com (A.P.); dr.iacobstefan@yahoo.com (S.I.); roatacristianene@gmail.com (C.E.R.); velenciucn@gmail.com (N.V.); gdimofte@gmail.com (G.D.); 2 Department of Surgery, University of Medicine and Pharmacy “Grigore T. Popa”, 700115 Iasi, Romania; 3 Radiotherapy Department, Regional Institute of Oncology (IRO), 700483 Iasi, Romania; irina_iftincai@yahoo.com (I.I.); andreea.marinca@gmail.com (A.M.); 4 “TRANSCEND” Centre for Fundamental Research and Experimental Development in Translational Medicine, Regional Institute of Oncology (IRO), 700483 Iasi, Romania; iuliuic@gmail.com

**Keywords:** rectal cancer, radiotherapy, DNA, neoadjuvant therapy, prognosis

## Abstract

*Background and Objectives:* The objective of this study was to investigate quantitative changes in cell-free DNA (cfDNA) found in the bloodstream of patients with locally advanced rectal cancer who received neoadjuvant long-course chemoradiation, assuming a change in DNA fragments release during therapeutic stress. *Materials and Methods:* This was a prospective observational study that involved 49 patients who had three distinct pathologies requiring neoadjuvant chemoradiation: 18 patients with breast cancer, 18 patients with cervical cancer, and 13 patients with rectal cancer. Both breast cancer and cervical cancer patients were used as a control groups. Breast cancer patients were used as a control group as irradiation targeted healthy tissue after the tumor resection (R0), while cervical cancer patients were used as a control group to evaluate the effect of chemoradiation regarding cfDNA in a different setting (squamous cell carcinomas) and a different tumor burden. Rectal cancer patients were the study group, and were prospectively evaluated for a correlation between fragmentation of cfDNA and late response to chemoradiation. Blood samples were collected before the initiation of treatment and after the fifth radiation dose delivery. cfDNA was quantified in peripheral blood and compared with the patients’ clinicopathological characteristics and tumor volume. *Conclusion:* Thirteen patients with locally advanced rectal cancer (T3/T4/N+/M0) were included in the study, and all of them had their samples analyzed. Eight were male (61.54%) and five were female (38.46%), with an average age of 70.85 years. Most of the patients had cT3 (53.85%) or cT4 (46.15%) tumors, and 92.31% had positive lymph nodes (N2–3). Of the thirteen patients, only six underwent surgery, and one of them achieved a pathological complete response (pCR). The mean size of the tumor was 122.60 mm^3^ [35.33–662.60 mm^3^]. No significant correlation was found between cfDNA, tumor volume, and tumor regression grade. cfDNA does not seem to predict response to neoadjuvant chemoradiotherapy and it is not correlated to tumor volume or tumor regression grade.

## 1. Introduction

Recent advancements in the multimodal treatment of locally advanced rectal cancer have resulted in impressive rates of clinical complete response (cCR) [1,2]. In a comprehensive systematic review conducted by Hartley et al., involving a total of 3157 patients, the overall pathological complete response (pCR) rate was estimated at 13.5% [3]. Notably, consistently higher response rates have been associated with high-dose radiotherapy [4]. The Rectal Cancer and Preoperative Induction Therapy Followed by Dedicated Operation (RAPIDO) trial, in particular, demonstrated that patients undergoing Total Neoadjuvant Therapy (TNT) achieved a significantly higher pCR rate (28.4%) compared to those receiving standard radiochemotherapy (14.3%, *p* < 0.0001) [5].

Furthermore, a strategy centered on watchful waiting (W&W), with the aim of organ preservation, has emerged as a viable option for patients who achieve clinical complete response (cCR) following neoadjuvant (chemo)radiotherapy [6]. This W&W approach, initially proposed by Habr-Gama, has received recent support from the International Watch and Wait Database, which reported a 2-year cumulative local regrowth incidence of 25.2%. In contrast, it is worth noting that even the pathologic complete response (pCR) rate, classified as Dworak TRG 4, reached as high as 28.4%. In the same study, the favorable responders, categorized as Dworak TRG 2–3, constituted 52.7% of the cases, while the poor-to-non-responders, classified as Dworak TRG 0–1, comprised 38.2% [7].

The current standard approach for locally advanced rectal cancer (T3/T4 N+) involves long-course radiotherapy, typically utilizing conventionally fractionated radiotherapy. This entails administering doses of 180 to 200 centi-Gray per fraction, delivered in 25 to 28 daily fractions (five days per week), resulting in a cumulative dose ranging from 4500 to 5040 centi-Gray. Concurrently, chemotherapy is administered, with the most commonly used cytostatic agents being capecitabine at a dose of 825 mg/m^2^ twice daily or 5-fluorouracil at a dose of 1200 mg/m^2^ daily [2,5]. Surgery is usually scheduled for 4 to 12 weeks following the conclusion of radiotherapy, typically in the sixth or seventh week, resulting in a total treatment duration of 3–4 months. Importantly, this timeframe does not account for waiting periods associated with initiating radiation therapy and surgical treatment.

The next crucial step in modern rectal cancer treatment is to establish standardized biomarkers [8] capable of estimating response rates and defining individualized neoadjuvant protocols [9]. Currently, there is no universally accepted “gold standard” for distinguishing between individuals who respond positively to treatment and those who do not. The aspiration is to identify specific biomarkers that can assist in differentiating potential good responders, who would benefit from radiochemotherapy, from those who may not respond as favorably. Such differentiation can play a pivotal role in sparing the latter group from enduring an extended treatment regimen with limited potential for tumor size reduction or downstaging, thereby mitigating the risk of undue toxicity and further tumor progression.

More recently, the selection of mismatch repair-deficient (dMMR) [8] patients for neoadjuvant immunotherapy with PD-1 blockers has shown impressive results in a small cohort of cases [2,10,11]. Trials such as NICHE (pCR 13/32—69%) and PICC (pCR 4/17—88%) have demonstrated the potential of this approach. DNA mismatch repair deficiency is already an established predictor, as is Immunoscore [12], which has been shown to predict survival and response rates to neoadjuvant therapy. In addition to tumor biopsies, readily available biomarkers harvested from peripheral blood [13] are under intense study and proposed as simpler alternatives. Immune cell ratios, CRP (C-reactive protein), and CD8+ T cells have all been described as potential predictors, although results have been conflicting [12].

Another potential biomarker under consideration is cell-free DNA (cfDNA). CfDNA comprises DNA fragments ranging in length from 50 to 200 base pairs. These fragments are released by cells and enter the bloodstream, typically as a result of apoptosis or necrosis [14]. The term “circulating tumor DNA” (ctDNA) specifically refers to cfDNA originating from cancer cells in cancer patients [15]. Mandel and Metais were the pioneers who first described cfDNA in 1948 [16]. It has recently gained recognition as a promising biomarker, particularly for diagnosing advanced malignancies [13]. Individuals with rectal cancer can exhibit cfDNA levels up to 50 times higher compared to those in a healthy state. During neoadjuvant therapy, these levels are expected to increase due to tumor necrosis [17,18].

Numerous studies have produced promising results regarding the utility of cfDNA in diagnosing, monitoring, and prognosticating rectal cancer. Both Zitt et al. and Agostini et al. have concluded that responders to treatment exhibit a significant reduction in circulating DNA, whereas non-responders tend to experience a notable increase in circulating DNA levels following radiochemotherapy [19,20]. Truelsen et al. have proposed circulating cell-free DNA as a predictor of pathological complete response, providing potential value in monitoring patients with a complete clinical response within watch-and-wait (W&W) strategies [7].

For the above reasons, our aim was to investigate quantitative changes in cfDNA found in the bloodstream of patients with locally advanced rectal cancer who received neoadjuvant long-course chemoradiation, assuming changes in DNA fragment release during therapeutic stress, particularly early after the fifth session of radiation.

## 2. Materials and Methods

### 2.1. Design and Setting

This was a single-center, observational, prospective study of three groups of patients who had three distinct cancers requiring neoadjuvant chemoradiation: breast cancer, cervical cancer, and rectal cancer. All patients underwent standard oncological work-up and management based on multidisciplinary meetings. All patients were treated and followed at our institution. Informed consent was gained for each patient included in the study. This study was approved by our Institutions Ethics Committee (REGISTRATION NUMBER. 227/26 August 2019).

Rectal cancer patients were the study group, and were prospectively evaluated for a correlation between fragmentation of cfDNA and late response to chemoradiation. Breast cancer patients were used as a control group as irradiation targeted healthy tissue after the complete tumor resection, while cervical cancer patients were used as a control group to evaluate the effect of chemoradiation on cfDNA in a different histological type and a different tumor burden.

### 2.2. Inclusion and Exclusion Criteria

Patients with confirmed histology and full oncological work-up were included. Only patients with curative intent were included. Patients with other synchronous cancers were excluded. Patients with previous malignancies were excluded.

### 2.3. Experimental Protocol

Blood samples were collected before the initiation of treatment and after the 5th radiation dose delivery. The need for an early prediction of response cannot be overemphasized and the 5th dose was chosen because surgical treatment can still be initiated, in patients considered non-responders, within a week after the 5th dose, while continuation of treatment may only result in toxicity and prolong by 2 months the time for surgical procedure. Two samples were collected from each patient, one before radiotherapy and the other after the fifth dose. Within four hours of collection, DNA from the plasma was extracted using the Cobas cfDNA Sample Extraction Kit^®^ (Roche Diagnostic GmbH, Mannheim, Germany) and stored at a temperature of −20 °C. Once all the samples were collected, we measured the amount of cfDNA using the Agilent 2100 Bioanalyzer (Agilent Technologies, Waldbroan, Germany) with High Sensitivity DNA Kit^®^ ( Agilent Technologies, Waldbroan, Germany). The Agilent 2100 Bioanalyzer system is an established microfluidics-based automated electrophoresis solution for the sample quality control of biomolecules.

After obtaining the concentrations of cfDNA from all the samples, we proceeded to input the data into XLSTAT for a comprehensive analysis. This analysis encompassed the examination of differences (i.e., cfDNA concentration after the 5th radiation session minus cfDNA concentration before radiation) and ratios (i.e., cfDNA concentration after the 5th radiation session divided by cfDNA concentration before radiation) between each sample collected before radiation and after the 5th radiation session of the same patient. For these data sets, we computed the range (defined as the difference between the smallest and largest values), the average (which included the average cfDNA concentration before radiation, after radiation, as well as the averages of the calculated differences and ratios), and variance (calculated using the Variance.P function). Additionally, employing the software, we generated linear regression plots and Box and Whisker graphs to facilitate a comparative analysis between the control group (comprising cases of breast and cervical cancer) and the target group (consisting of rectal cancer cases).

Response to chemoradiation was assessed 8 weeks after completion of radiation therapy through pelvic MRI. Data on tumor volume were collected, including gross tumor target volume (GTV) and planning target volume (PTV) from the archive of treatment plan from the radiotherapy department. Tumor response was extracted from the pathological response rating (Dworak grading). Descriptive variables are reported as percentages, mean, and range for clinicopathologic characteristics and tumor measurements. The association between cfDNA distribution and tumor volume and tumor regression grade (TRG) was evaluated using the Pearson correlation coefficient test. A *p* value < 0.05 was considered statistically significant. XLSTAT software (Version 2309 Build 16.0.16827.20014) was used for statistical analysis.

## 3. Results

### 3.1. Patient Characteristics

This prospective study investigated 98 samples (from 49 patients). Specifically, all 18 patients with breast cancer had their samples analyzed, along with 18 patients from the cervical cancer group and 13 patients from the rectal cancer group.

**Figure 1:**
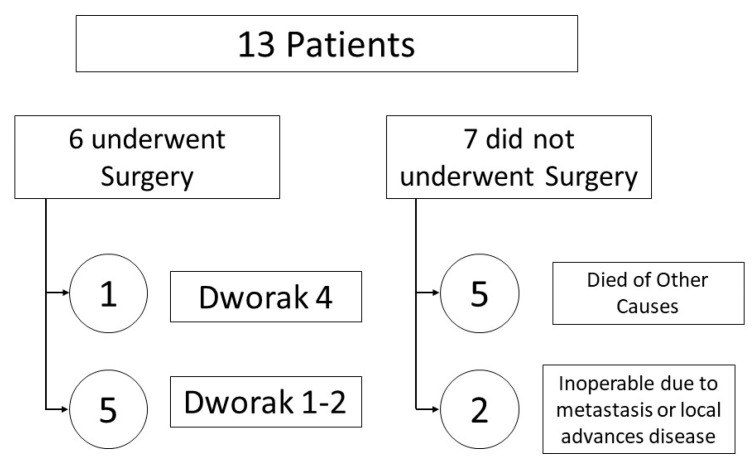
Diagram depicting management and response in the thirteen rectal cancer patients.

Thirteen patients with locally advanced rectal cancer (T3/T4/N+/M0) were included in the study, and all of them had their samples analyzed; eight were male (61.54%) and five were female (55.56%), with an average age of 70.92 years. Most patients had cT3 (53.85%) or cT4 (46.15) tumors, and 92.31% had positive lymph nodes (N1–2). The disease stage was IIIc for nine patients, IIIb for three patients, and IIb for one patient. Of the thirteen patients, only six underwent surgery, and one of them achieved a pathological complete response (pCR). Two of the seven patients who did not have surgery were deemed inoperable due to metastasis or advanced disease and five patients died of other causes (Figure 1). All patients that received the standard concurrent chemoradiotherapy protocol were reevaluated after 8 weeks, and underwent surgical resection with total mesorectal excision (TME). Of six patients who underwent surgical resection, five patients (Dworak 1–2) showed poor response and one patient showed pathological complete response (Dworak 4). Table 1 summarizes the findings.

### 3.2. Tumour Measurements

Thirteen patients were analyzed with regard to the mean size of the gross target volume (GTV), which measured 122.60 mm^3^ (ranging from 35.33 mm^3^ to 662.6 mm^3^). The planning target volume (PTV) for these patients was determined to be 1839.75 mm^3^, with a range of 1122.18 mm^3^ to 2910 mm^3^. To further investigate the correlation between tumor volume and tumor regression grade, a subgroup was established for patients who had undergone surgery, totaling six individuals. In this subgroup, the mean size of the gross target tumor volume was 75.25 mm^3^, ranging from 60.92 mm^3^ to 86.54 mm^3^. Additionally, the planning target volume (PTV) was calculated as 2067.27 mm^3^, ranging from 1463.40 mm^3^ to 2910 mm^3^ (Table 2).

### 3.3. Quantification of cfDNA

In total, 98 samples were gathered and examined from 49 patients, (including 18 from the breast cancer group, 18 from the cervical cancer group, and 13 patients from the rectal cancer group).

**Figure 2:**
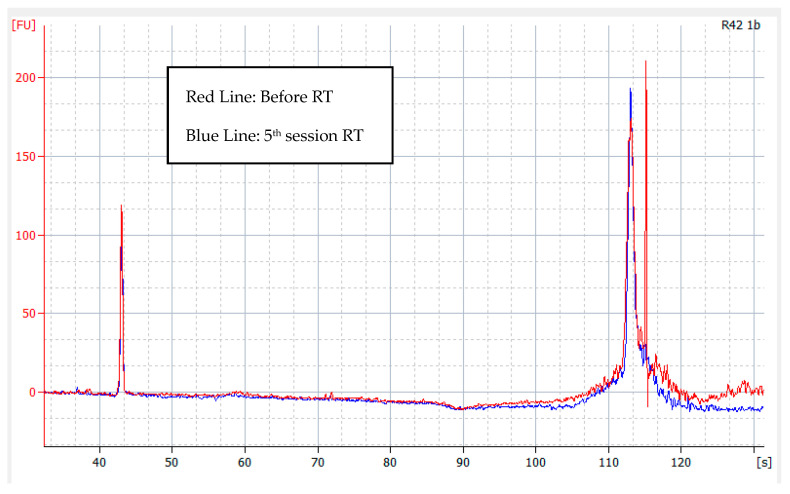
Example of result for cfDNA before and after 5th session of radiotherapy.

Figure 2 displays an illustration of the outcome produced by utilizing the Agilent 2100 Bioanalyzer along with the High Sensitivity DNA Kit. The *X*-axis illustrates the concentration detected in seconds (s), while the *Y*-axis represents the concentration in picograms per microliter (pg/µL). The final result was computed through automated calculations using the Agilent 2100 Expert software(ver. B.02.08.SI648 [SR 1]) provided by Agilent 2100 Bioanalyzer. The kit is designed to accurately quantify and determine the size of DNA fragments and smears that fall within the range of 50 to 7000 bp. The red line represents the result prior to radiotherapy, while the blue line represents the outcome after the fifth dose of radiotherapy. We evaluated the area under the curve, which allowed us to estimate the total quantity of DNA fragments between the two collections and determine the ratio and differences between them.

### 3.4. Distribution of cfDNA

**Figure 3:**
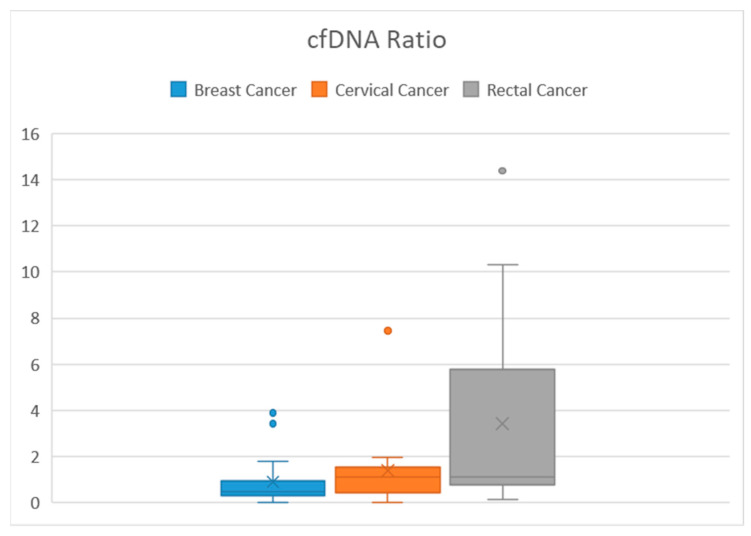
Box and Whisker plot of cfDNA ratio between samples.

Table 3 displays the mean value and variability of each sample group. For the target group (consisting of thirteen rectal cancer patients), the mean volume of cfDNA fragments collected from plasma was 455.45 pg/μL (with a range of 70.90–1013.80 pg/μL) before radiotherapy, and the mean volume on the 5th day of radiotherapy was 816.92 pg/μL (with a range of 72.50–3336.30 pg/μL). In the breast cancer control group, 18 samples were collected both before and on the 5th day of radiotherapy. The median value before treatment was 1494.92 pg/μL (with a range of 133.90–10,241.20 pg/μL), while the mean on the 5th day of radiotherapy was 1049.72 pg/μL (with a range of 62.3–6196.50 pg/μL). For cervical cancer patients, the mean concentration of cfDNA before radiotherapy was 3063.26 pg/μL (with a range of 300.80–22,660.00 pg/μL), and the mean value on the 5th day was 2192.03 pg/μL (with a range of 176.80–13,106.90 pg/μL). Figure 3 depicts the Box and Whisker plot of all samples. The overall data exhibited several outliners, and no discernible pattern or correlation was observed.

### 3.5. Regression and Pearson Correlation Coefficient Test

**Figure 4:**
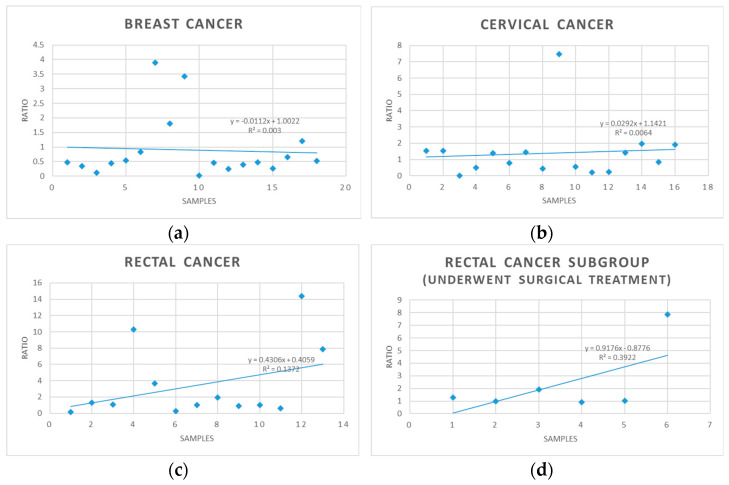
cfDNA ratio regression graph in all three groups of patients: (**a**) breast cancer group, (**b**) cervical cancer group, (**c**) rectal cancer group, (**d**) rectal cancer subgroup (underwent surgical treatment).

Figure 4 displays a regression graph illustrating the cfDNA ratio within all three patient groups. Notably, rectal cancer (r^2^ = 0.1372) and cervical cancer (r^2^ = 0.064) show a positive regression trend, while breast cancer exhibits a negative regression, with an r^2^ value of 0.003. It is important to emphasize that the Pearson test did not identify any significant correlations among cfDNA levels (measured both before and on the 5th day of radiotherapy), tumor volumes (GTV, PTV), and tumor regression grades (TRG), as outlined in Table 4.

## 4. Discussion

In this pilot study, we successfully demonstrated the extraction and standardized quantification of cfDNA from peripheral blood. However, in this specific experiment, a significant correlation was not observed between cfDNA levels and tumor response. This raises questions about the predictive role of cfDNA, which in turn prompts the need for further research and debate concerning the timing and methodology of cfDNA quantification, as its distribution may vary during neoadjuvant therapy.

As previously mentioned, a minor quantity of circulating free DNA (cfDNA) is typically detected in healthy individuals. Quantitative studies have shown that in healthy individuals, cfDNA concentrations usually fall within the range of 0–100 ng/mL of blood, with an average of 30 ng/mL, whereas in the blood of cancer patients, cfDNA concentrations exhibit a broader variation, ranging from 0 to 1000 ng/mL, with an average of 180 ng/mL [1,14,18].

Numerous studies have generated promising findings regarding the utility of circulating cell-free DNA (cfDNA) in monitoring the response to radiochemotherapy in patients with locally advanced rectal cancer. In a study conducted by M. Zitt et al., responders exhibited a reduction in cfDNA to 2.2 ng/mL (with a range of 1.5 to 2.9 ng/mL), while non-responders showed an increase to 5.1 ng/mL (with a range of 3.8 to 10.3 ng/mL) (*p* = 0.006) [19].

Furthermore, a separate investigation led by W. Sun et al. revealed a significant decline in the concentration of 400-base pair fragment DNA following chemoradiotherapy, particularly within the group exhibiting a favorable treatment response (TRG 0, 1, 2 group, *p* = 0.17; TRG 3, 4 group, *p* < 0.01) [21]. Additionally, another study demonstrated a noteworthy association between the levels of cell-free DNA and the presence of recurrent disease. Patients with recurrent disease displayed a median level of 13,000 copies/mL, in contrast to the 5200 copies/mL observed in non-recurrent patients (*p* = 0.08). This investigation also established a correlation between the total cell-free DNA levels and both the pathological stage and nodal involvement [22].

In our study, we established a control group comprising breast cancer patients undergoing adjuvant radiation therapy (RT). Our hypothesis was that following neoadjuvant therapy and mastectomy, most tumor cells would be eliminated. Adjuvant RT was then administered to irradiate healthy breast tissue with minimal residual tumor cells. Our study highlights a distinction between the breast cancer group, characterized by a limited tumor burden, and the cervical cancer group, which exhibits a more substantial tumor burden and is known for its favorable response to chemoradiation.

Before radiation therapy, cfDNA levels in the breast cancer group ranged from 133.90 to 10,241.20 pg/μL, with an average of 1494.92 pg/μL. In the cervical cancer group, the range was from 300.80 to 22,660.00 pg/μL, with an average of 3063.26 pg/μL. On the 5th day post-treatment, cfDNA levels in the breast cancer group ranged from 62.30 to 6196.50 pg/μL, while the cervical cancer group exhibited a wider range and a higher average (range: 176.80–13,105.90 pg/μL, average: 2192.03 pg/μL).

The objective of our study was to investigate quantitative variations in circulating free DNA (cfDNA) in patients diagnosed with locally advanced rectal cancer who underwent neoadjuvant long-course chemoradiation. We hypothesize that there may be changes in DNA fragment release during therapeutic stress. To achieve our goal, we established two control groups representing the upper and lower limits of cfDNA levels. Our aim is to differentiate between patients who will respond favorably to neoadjuvant long-course chemoradiation and those who will not. To accomplish this, we worked to establish a correlation between the difference in cfDNA levels before and on the 5th day of radiation treatment and the tumor regression grade (TRG) determined through pathological examination of surgically removed tumor specimens.

Unfortunately, among the initial cohort of thirteen patients who initiated neoadjuvant radiochemotherapy, only six patients ultimately underwent surgical resection. Notably, one patient exhibited a complete pathological response classified as Dworak 4, while the remaining patients demonstrated suboptimal responses, with three patients categorized as Dworak 1 and two patients as Dworak 2. Among the seven patients who did not undergo surgery, three were deemed ineligible for surgical intervention due to the presence of metastasis or locally advanced disease, while the unfortunate outcome was observed in four patients who succumbed to causes unrelated to their oncological condition.

Truelsen et al. from Elsevier propose that circulating cell-free DNA (cfDNA) can serve as a valuable biomarker and complementary tool to imaging in identifying candidates for a Wait and Watch (W&W) strategy among patients who have achieved a clinical complete response (cCR). They suggest that patients characterized as “cfDNA responders” may have an association with achieving a pathological complete response (pCR). In their investigation, cfDNA samples were collected at baseline, during the midpoint of therapy, and at the conclusion of treatment [7].

In contrast, our study adopted a different approach by collecting cfDNA samples before the initiation of radiation therapy and after the fifth session of radiation. The primary objective was to acquire early results that could assist in distinguishing patients who are unlikely to respond favorably to chemoradiation therapy. This approach sought to identify poor or non-responders at an early stage and to mitigate treatment-related toxicity [23] and reduce the extended waiting period, typically spanning 6–8 weeks after neoadjuvant therapy, before commencing surgical intervention.

In the subgroup of our study (Table 2 and Table 5), in one patient who achieved a pathological complete response (pCR) before undergoing radiation therapy, the cfDNA level was measured at 137.80 pg/μL before treatment and a slight increase to 175.70 pg/μL after the fifth session, resulting in a difference of 37.90 pg/μL and a ratio of 1.28. For patients classified as Dworak 1 and 2, the cfDNA levels ranged from 70.90 pg/μL to 828.50 pg/μL before radiation therapy, with an average of 470.64 pg/μL. The average difference in cfDNA levels was 210.14 pg/μL, and the ratio was 2.54. Among patients who did not undergo surgery due to the presence of metastasis or locally advanced disease, baseline cfDNA levels ranged from 136.20 pg/μL to 327.50 pg/μL, with an average of 231.85 pg/μL. After the fifth session of radiation therapy, cfDNA levels ranged from 359.50 pg/μL to 1957.2 pg/μL, with an average of 1158.35 pg/μL. The average ratio was 7.73 and the average difference was 926.5 pg/μL. Noticeably, higher differences and ratios were observed in patients who were not eligible for surgical treatment. However, due to the limited size of our cohort, our study was unable to establish statistically significant results.

Cellular necrosis and apoptosis represent the primary sources of circulating cell-free DNA in plasma following radiation. Another plausible hypothesis involves exploring the correlation between cfDNA, tumor volume and the planning target volume. Several studies have yielded positive findings regarding the relationship between cfDNA levels and tumor size. All of our locally advanced rectal cancer patients underwent long-course radiotherapy (50.4 Gy/28F/5w), either with or without concomitant capecitabine at 850 mg/m^2^ twice daily (BID). By maintaining this control variable, we aimed to establish a positive association between tumor size (GTV), planning target volume (PTV), and cfDNA quantity after the initiation of radiation.

Regrettably, despite all patients experiencing a reduction in tumor size following neoadjuvant chemoradiotherapy, no clear relationship between cfDNA and gross tumor volume (GTV), planning target volume (PTV), and tumor regression grade (TRG) was discerned [24]. This lack of correlation may be attributed to the insufficient number of samples and the timing of sample collection. It is conceivable that, on the 5th day post-radiotherapy, a substantial quantity of cfDNA has not yet been released, necessitating a more extended monitoring period to capture a significant release of cfDNA during and after the completion of radiotherapy. This consideration aligns with evidence indicating that tumors continue to respond to radiotherapy for at least two months after treatment completion. Other studies have taken a weekly sampling approach during radiotherapy, correlating the cumulative cfDNA levels with TRG or distinguishing between complete responders and non-responders [25]. Similarly, Truelsen et al. collected cfDNA samples at the onset, midpoint, and conclusion of radiotherapy, subsequently comparing the mean values of these samples using ROC curve analysis in relation to TRG [7].

This study recognizes the need for further research to address the current limitations of cfDNA analysis and enhance its prognostic utility in rectal cancer. It underscores the importance of conducting larger, more comprehensive studies to validate these findings and optimize cfDNA analysis as a prognostic tool for rectal cancer.

## 5. Conclusions

cfDNA does not seem to predict response to neoadjuvant chemoradiotherapy and it is not correlated to tumor volume or tumor regression grade.

## Figures and Tables

**Table 1:** Characteristics of included rectal cancer patients.

Clinical Variable	No. (%)
**Sex:**	
Male	8 (61.54)
Female	5 (38.46)
**Age:**	
<65	3 (23.07)
>65	10 (46.15)
**Clinical TNM Staging**	
Tumor	
cT3	7 (53.85)
cT4	6 (46.15)
Node	
cN0	1 (7.69)
cN1	3 (23.08)
cN2	9 (69.23)
Metastasis	
cM0	13 (100)
cM1	0 (0)
**Clinical AJCC Staging**	
IIb	1 (7.6)
IIIa	0 (0)
IIIb	3 (23.07)
IIIc	9 (69.23)
**Management**	
Operated	6 (46.1)
Not operated	7 (53.8)
Metastasis or locally advanced	2 (28.57)
Other causes	5 (71.42)
**Pathology Staging**	
Tumor	
ypT0	1(16.67)
ypT1	0 (0)
ypT2	2 (33.33)
ypT3	3 (50.00)
Node	
ypN0	5 (83.33)
ypN1	1 (16.67)
**Pathological Response**	
Dworak TRG system	
1 (poor response)	3 (50.00)
2 (poor response)	2 (33.33)
3 (good response)	0 (0)
4 (Complete Responds)	1 (16.67)

**Table 2:** Tumor measurements in the rectal cancer cohort that underwent surgical treatment. Key: GTV, gross target volume; PTV, planning target volume; cTN, clinical tumor node staging; ypTN, pathological tumor node staging; TRG, tumor regression grade.

Sample	GTV (mm^3^)	PTV (mm^3^)	cTN	ypTN	TRG
R42	69.87	2177.66	cT3N2	ypT0N0	4
R50	86.54	1502.27	cT3N1	ypT3N0	1
R51	66.73	1633.77	cT3N1	ypT3N0	1
R52	60.92	1463.4	cT3N2	ypT2N0	2
R53	83.79	2910.00	cT3N1	ypT2N1	2
R61	83.63	2716.5	cT4N2	ypT3N0	1
Average	75.25	2067.27			

**Table 3:** Average and variance of cfDNA.

		Range	Average	Variance
Rectal Cancer	Before:	70.90—1013.80	455.45	116,860.18
	5th:	72.50–3336.30	816.92	789,032.72
	Difference:	−741.80–2427.00	361.48	737,937.48
	Ratio:	0.13–14.37	3.42	18.93
Breast Cancer	Before:	133.90–10,241.20	1494.92	5,206,590.66
	5th:	62.30–6196.50	1049.72	2,763,380.14
	Difference:	−10,083.00–4604.60	−445.20	7,669,628.27
	Ratio:	0.02–3.89	0.90	1.12
Cervical Cancer	Before:	300.80–22,660.00	3063.26	28,769,505.87
	5th:	176.80–13,105.90	2192.03	12,388,219.88
	Difference:	−22,203.40–11,347.80	−871.23	40,621,472.94
	Ratio:	0.02–7.45	1.39	2.82

Before: before radiotherapy; 5th: after 5th day of radiotherapy; Difference: difference between 5th day of radiotherapy and before radiotherapy; Ratio: ratio between 5th day of radiotherapy and before radiotherapy.

**Table 4:** Pearson correlation coefficient between cfDNA, tumor volume and TRG. (Key: cfDNA, cell-free DNA; pre-RT, before radiotherapy; GTV, gross target volume; PTV, planning target volume; TRG, tumor regression grade; Difference, difference between cfDNA after the fifth session of RT and cfDNA before RT).

	Pearson Correlation Coefficient (r)	*p* Value
cfDNA pre-RT—PTV	0.05	0.92
cfDNA pre-RT—GTV	0.49	0.31
Difference—GTV	0.04	0.94
cfDNA after 5th session RT—GTV	0.41	0.42
cfDNA after 5th session RT—PTV	0.41	0.92
Difference—TRG	0.50	0.31

**Table 5:** Data between cfDNA (pg/μL), tumor volume (mm^3^) and TRG. Key: RT, radiation therapy; GTV, gross target volume; PTV, planning target volume; TRG, tumor regression grade.

Sample	Pathological Response	cfDNA before RT	cfDNA 5th Day RT	Difference between cfDNA 5th Day RT and cfDNA before RT	GTV	PTV
R42	Complete respond TRG 4	137.80	175.70	37.90	68.87	2177.66
R50	Dworak TRG1	828.50	818.80	−9.70	86.54	1502.27
R51	Dworak TRG 1	595.70	1150.10	554.40	66.73	1633.77
R52	Dworak TRG2	80.60	72.50	−8.10	60.92	1463.40
R53	Dworak TRG2	777.50	804.00	26.50	83.79	2910.00
R61	Dworak TRG 1	70.90	558.50	487.60	83.63	2716.50

## Data Availability

The data that support the findings of this study are available on request from the corresponding author, S.M.
